# Detection and complete genome sequence analysis of human adenovirus in children with acute diarrhea in Yunnan, China, 2015–2021

**DOI:** 10.1007/s00705-023-05950-z

**Published:** 2024-01-23

**Authors:** Yihui Cao, Jinghui Yang, Nan Li, Ruixian Zhang, Lili Jiang, Xiaofang Zhou, Yibin Xiang, Jianping Cun, Enfa Qiao

**Affiliations:** 1 Yunnan Center for Disease Control and Prevention, Kunming, 650022 China; 2 The First People’s Hospital of Yunnan Province, Kunming, 650032 China

**Keywords:** Acute diarrhea, Viral pathogen, Human adenovirus, Southwest China, Surveillance

## Abstract

The aims of this study were to determine the distribution and prevalence of gastroenteritis caused by human adenovirus (HAdV) in children in Yunnan province, China, in 2015–2021 and to identify preventive measures that can be taken to reduce morbidity and mortality in children.HAdV is a significant agent of diarrhea in children, but limited data are available regarding the epidemiology and genetic diversity of HAdV in children with diarrhea in Yunnan province, China. A total of 1754 fecal samples were subjected to real-time RT-PCR to detect and quantify HAdV. Positive samples were further analyzed using next-generation sequencing (NGS), and epidemiological data were analyzed as well.1754 patients with diarrhea were enrolled, of which 1041 were male and 713 were female (M:F ratio: 1.46). Seventy-two stool samples out of 1754 (4.10%) were positive for HAdV. The detection rates of all age groups varied from 2.50–4.78%. The highest incidence of HAdV was observed in children under 2 years of age, especially in children 12–24 months-old. From 2015–2021, the annual detection rate ranged from 1.62–12.26%. HAdV was detected throughout the year, but with marked seasonality. Children were most likely to be positive for HAdV in June and November. We detected HAdV in 15.53% (16/103) of samples collected in June and in 8.19% (14/171) of those collected in November. The entire viral genome was successfully sequenced for 13 of the 72 HAdV-positive samples, and 76.92% (10/13) of these were classified as genotype F41 and 23.08% (3/13) were classified as genotype C2.ConclusionsIn Yunnan province, children of all ages are susceptible to HAdV infection, but there has been marked variation in the yearly prevalence. The highest rate of HAdV detection was in June, followed by November. Priority should be given to disease prevention over the development of targeted antiviral therapies, and effective vaccines for preventing HAdV diarrhea are needed. It is also important to establish a surveillance system to collect relevant clinical and epidemiological data quickly in order to assess the potential risk of HAdV infection in children and to identify epidemic strains for the development of effective vaccines.

## Introduction

Acute diarrhea is one of the major causes of morbidity and mortality among young children, especially in developing countries [1]. It often poses a heavy social and economic burden [2–5]. Although bacteria, viruses, and parasites can cause diarrhea, more than 50% of all diarrhea episodes have been found to be associated with viral pathogens [6, 7]. Human adenovirus (HAdV), rotavirus, and norovirus are the main diarrhea pathogens of young children [8, 9]. Outbreaks and sporadic cases of gastroenteritis caused by HAdV occur throughout the year and pose a major public health burden worldwide [10]. With the introduction of rotavirus vaccines in China, it seems certain that HAdVs will soon account for a larger proportion of the total cases of acute gastroenteritis.

HAdV is an nonenveloped virus belonging to the family *Mastadenovirus*. It has a linear, double-stranded DNA genome of 26–45 kb. To date, there are 111 recognized HAdV genotypes, which are classified into seven subgroups (A to G) (http://hadvwg.gmu.edu) according to their biophysical, biochemical, and genetic characteristics [11, 12]. HAdV can cause a wide variety of clinical disorders, including respiratory diseases, conjunctivitis, gastroenteritis, cystitis, cardiomyopathy, meningoencephalitis, hemorrhagic cystitis, and neurologic disease, some of which can be fatal [13, 14]. HAdV F40 and F41 are the main pathogens causing diarrhea, but other genotypes can also be involved, and the prevalence of HAdV genotypes varies in different regions [15, 16]. Although the importance of acute gastroenteritis as one of the major causes of morbidity and mortality is well recognized, few studies have been conducted to evaluate the role of HAdV in children with diarrhea in Yunnan.

The aim of this study was to investigate the epidemiology and genotype diversity of HAdV in children admitted to hospitals in Yunan with acute gastroenteritis and to estimate the incidence and examine the genetic diversity of HAdV strains circulating among children, with the hope of obtaining information that will be useful for devising intervention strategies.

## Materials and methods

### Patients and specimens

Acute diarrhea was defined as more than three episodes of abnormal (liquid, watery, mucous, or bloody purulent) stools per day or fewer than three abnormal stools per day with vomiting. A total of 1754 stool samples were collected from Children’s Hospital of Kunming and Yuxi Maternal and Child Care Service Center, Yunnan province, China, from January 2015 to December 2021. Demographic information, including sample collection date, age, and gender were also collected. Stool samples were stored at room temperature for no more than 4 hours before storage at -70°C.

### Detection of HAdV and next-generation sequencing (NGS)

All of the samples were tested by real-time PCR to detect HAdV. Stool samples were mixed with phosphate-buffered saline (PBS) to make a 10% suspension and then centrifuged at 12,000 × *g* for 3 minutes. Viral RNA was extracted from 200 µl of the resulting supernatant, using a Maxwell 16 Viral Total Nucleic Acid Purification Kit (ProMEGA, Madison, WI, USA) and an SAW-96 Automatic Nucleic Acid Extractor (Jiangsu Bioperfectus Technologies, China) in accordance with the manufacturer’s instructions.

HAdV was identified using a Real-Time PCR Diagnostic Kit for Rapid Detection of Adenovirus (XABT, China). The positive HAdV samples were sent to Shanghai Bojie Biotechnology Co., Ltd for NGS. The extracted nucleic acid from the HAdV-positive samples was amplified by PCR using an adenovirus genome enrichment kit (Shanghai Bojie Biotechnology Co., Ltd.). The amplification products were then purified, quantified, and sequenced using an Illumina Nextera XT Kit for deep sequencing on an Illumina MiSeq instrument with 2×300-base paired-end reads. The quality of the sequenced reads was assessed by using Fast QC. The Q30 value for the sequencing data was > 85%, and more than 90% of the sequencing reads reached Q30 (99.9% base call accuracy). The sequencing data volume of each sample was 1 Gb with 22–33 million reads, and the average sequencing depth was about 600x.

### Phylogenetic analysis

HAdV sequences were aligned with reference sequences using MEGA software (version11.0), and the neighbor-joining method was used to construct phylogenetic trees based on the complete genome sequence as well as the sequence of each of the three major capsid genes: hexon, fiber, and penton.

The analysis was performed in MEGA 11.0 using the Kimura 2-parameter method with 1000 bootstrap replicates. Thirteen sequences from in this study were submitted to the GenBank database with the accession numbers OP378826, OP555452, OP555453, OP555454, OP555461, OP555455, OP555462, OP555463, OP555456, OP555457, OP555458, OP555459, and OP555460.

### Statistical analysis

SPSS version 26.0 was used for statistical analysis. Differences in frequencies were evaluated using the chi-square test. A *p*-value < 0.05 was considered statistically significant.

## Results

A total of 1754 samples from children with acute diarrhea were collected from 2015 to 2021. Of these patients, 1041 were male and 713 were female (male-to -female ratio, 1.46), 83.69% (1468/1754) were younger than 2 years of age, and 92.36% (1620/1754) were younger than 3 years of age (Table 1). There were no significant differences in the detection rates in the various age groups, which ranged from 2.50–4.78%. The highest incidence of HAdV was observed in children between 1 and 2 years old (4.78%; 22/460).

Yunnan is located in southwestern China and has a mild climate all year round. Cases of acute diarrhea occurred throughout the year but exhibited a pronounced winter peak, lasting from October to January of the following year (n = 819). In the months of July to September, there were only 297 acute diarrhea cases. However, the highest rate of HAdV detection occurred in June (15.53%; 6/96), and the lowest was in December (0.36%; 1/281). Most of the patients with HAdV gastroenteritis (84.72%) were infants under 2 years of age. There were no statistical differences in the detection rates among the different age groups (*p* = 0.676) or between males and females (*p* = 0.671).

**Table 1:** HAdV infections in children with acute diarrhea

Variable	Number of cases	Number positive for HAdV	Percentage positive for HAdV (%)	*p*-value
Age in years				
≤ 0.5	511	16	3.13%	0.676
0.5 to 1	497	23	4.63%	
1 to 2	460	22	4.78%	
2 to 3	152	6	3.95%	
3 to 4	69	3	4.35%	
4 to 5	40	1	2.50%	
5 to14	25	1	4.00%	
Gender				
Male	1041	41	3.94%	0.671
Female	713	31	4.35%	
Total	1754	72	4.10%	

**Fig. 1:**
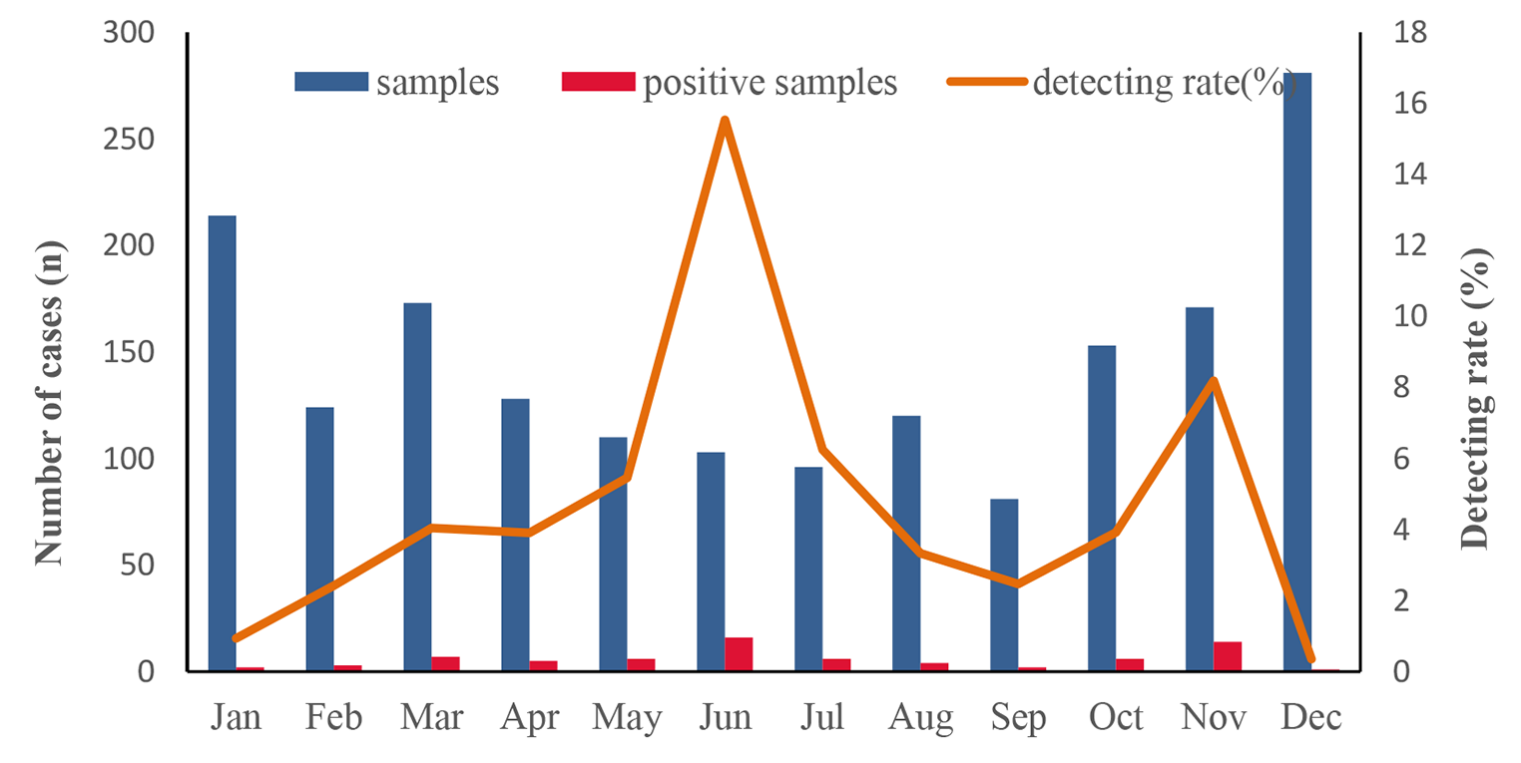
Human adenovirus cases and detection rate in different months, 2015–2021

HAdV was identified throughout the year. The incidence of HAdV infection was 4.10% (72/1754). There were two epidemic peaks of HAdV each year. The highest detection rate (15.53%; 16/103) was in June, followed by November (8.19%; 14/171).

Most of the total cases of diarrhea were in winter. The HAdV detection rate varied from 1.03–12.26% in 2015–2020. The prevalence of HAdV infection increased each year from 2015 to 2018: 1.62%, 2.62%, 3.96%, and 7.91% in 2015, 2016, 2017, and 2018, respectively. In 2019–2020, the number decreased sharply, but in 2021, the incidence of HAdV increased significantly. There were statistical differences in the positive detection rate in different years (*p* < 0.001).

**Table 2:** Diarrhea cases and HAdV detection rate in different years in Yunnan

Year	Number of cases of acute diarrhea (n)	Number positive for HAdV (n)	HAdV detection rate (%)
2015	308	5	1.62
2016	229	6	2.62
2017	227	9	3.96
2018	253	20	7.91
2019	390	4	1.03
2020	135	2	1.48
2021	212	26	12.26
Total	1754	72	4.10

The complete virus genome sequence was determined for 13 of the samples, 10 of which were identified as HAdV-F41, belonging to species F, and three of which belonged to the species C. HAdV-F41 and HAdV-C2 were the only genotypes identified in Yunnan province.

**Fig. 2:**
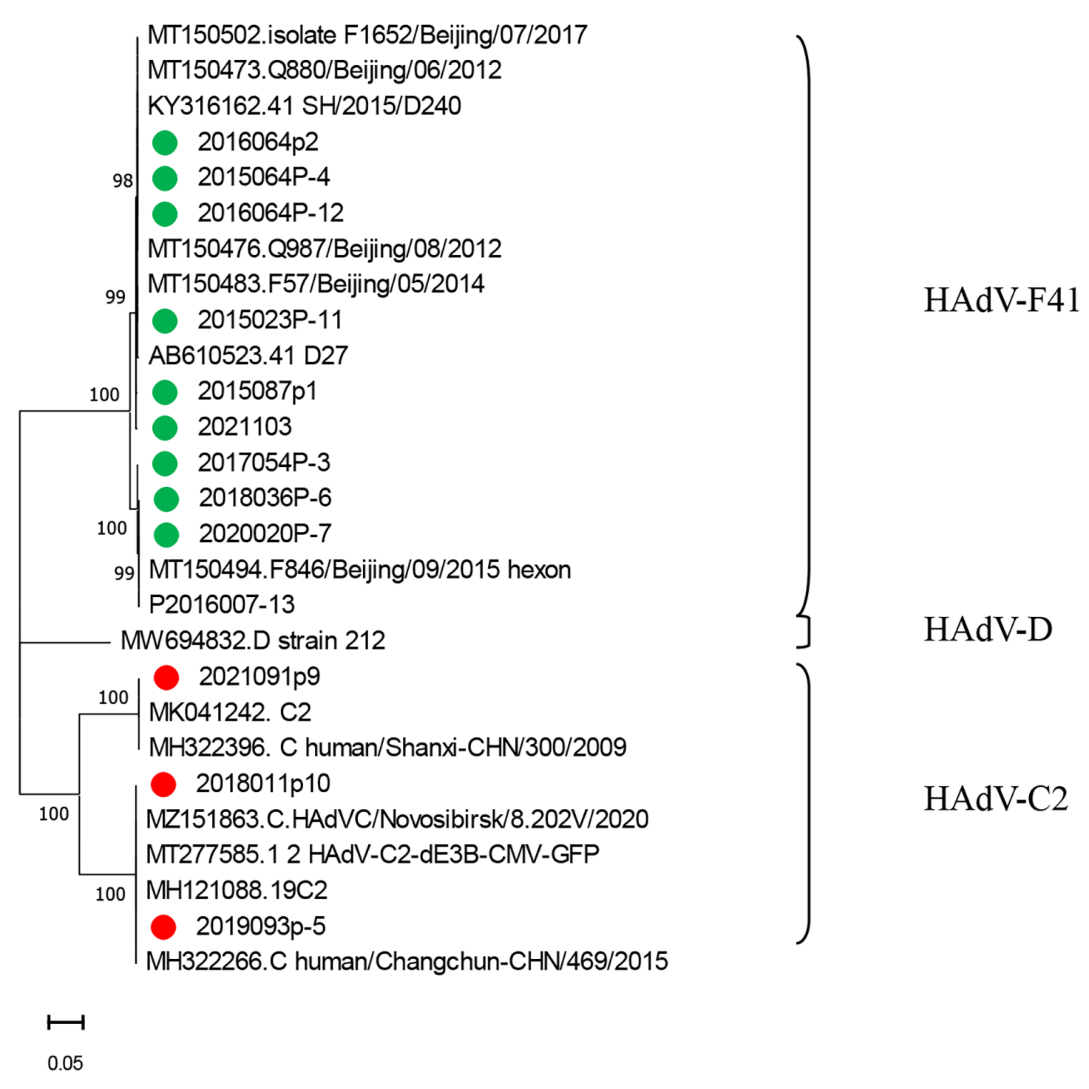
Phylogenetic tree based on nucleotide sequences of the HAdV hexon gene, constructed in MEGA version 11.0 by the neighbor-joining method with the Kimura 2-parameter model and 1000 bootstrap replications. HAdV-F41 strains from this study are indicated by a green circle (●). HAdV-C2 strains are indicated by a red circle (●). The scale indicates a genetic distance of 0.5.

**Fig. 3:**
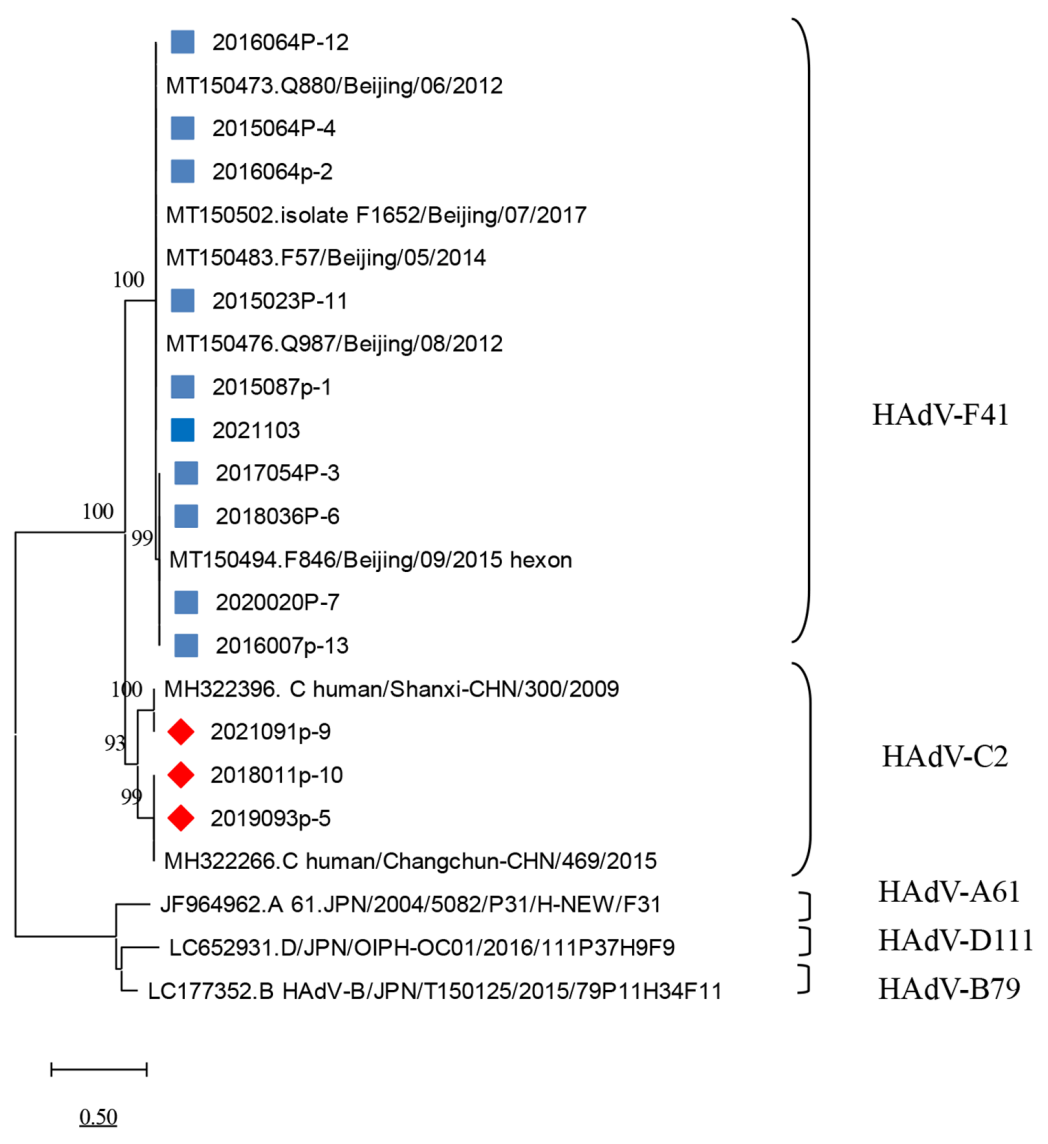
Phylogenetic tree based on nucleotide sequences of the HAdV penton gene, constructed in MEGA version 11.0 by the neighbor-joining method with the Kimura 2-parameter model and 1000 bootstrap replications. HAdV-F41 strains from this study are indicated by a blue square (■), and HAdV-C2 strains are indicated by a red diamond (◆). The scale indicates a genetic distance of 0.5.

**Fig. 4:**
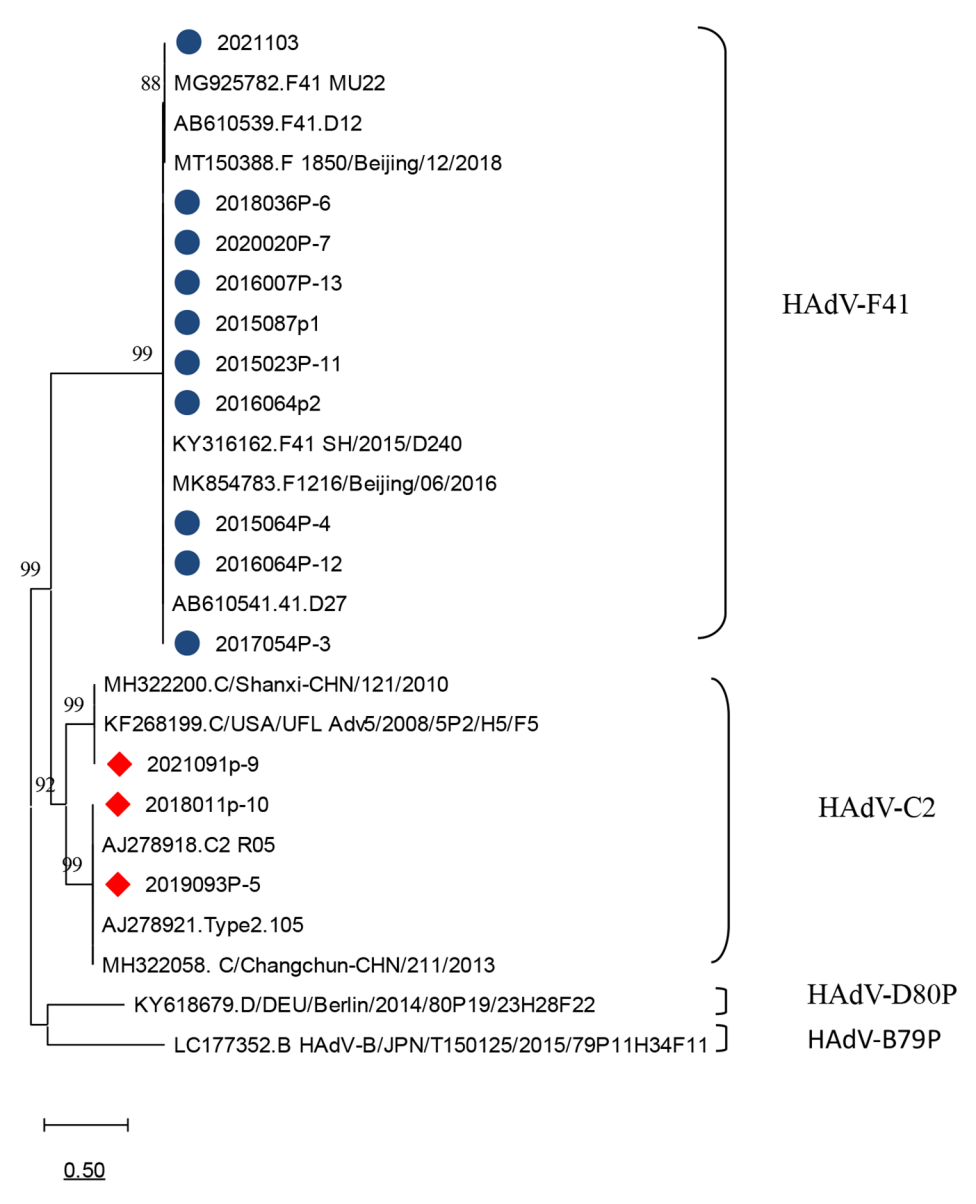
Phylogenetic tree based on nucleotide sequences of the HAdV fiber gene, constructed in MEGA version 11.0 by the neighbor-joining method with the Kimura 2-parameter model and 1000 bootstrap replications. Strains for this study are indicated by a blue circle (●) or a red diamond (◆).

## Discussion

Although it is well established that viruses are a major causes of acute gastroenteritis, prior to this study, there has been little information available about the burden of HAdV-related diarrhea among children in Yunnan Province, China. We conducted a 6-consecutive-year monitoring and epidemiology study of HAdV in association with acute gastroenteritis from January 2015 to December 2021 to assess HAdV prevalence and to examine the genetic characteristics of viruses obtained from hospitalized children with acute diarrhea. HAdV was detected in 72 samples from these patients, and the detection rate was 4.10%, which is similar to the rates reported previously in Shanghai [9], Guangzhou [17], and China as a whole [18].

Viral infection is a common cause of acute gastroenteritis [19], and HAdV is one of the common viral pathogens. HAdV species F types 40 and 41 are significantly associated with gastroenteritis, and type 41 appears to be more common than type 40 [20], although the prevalence and genotypes involved can differ among geographic regions. It has been reported that HAdV is the most important viral pathogen associated with gastrointestinal illnesses in some regions [21, 22]. In Thailand, HAdV subgroup C (40.6%) was found to be the most prevalent subgroup, followed by subgroup F [23]. Human enteric adenovirus F40/41 was a major cause of acute gastroenteritis in children in Brazil from 2018 to 2020 [11]. In China, among patients with acute gastroenteritis, HAdV-F41 has been reported to be the predominant type, followed by HAdV species B and C [17]. In the current study of HAdV in Yunnan province, 13 strains were sequenced, 10 of which were HAdV-F41 (76.92%) and three of which were HAdV-C2 (23.08%), HAdV-F40 and other types were not found, presumably because of the small number of sequences analyzed. HAdV-F41 was the main genotype associated with gastroenteritis. Our data are in line with what appears to be a global trend regarding the HAdV genotype distribution among gastroenteritis cases [24–26].

Wang et al. [27] reported that the prevalence of HAdV in China between 2009 and 2018 was 9.27%, based on a nationwide prospective surveillance of patients of all ages with acute diarrhea. From 2017 to 2019, the detection rate of HAdV was 4.44% in Chongqing, which is adjacent to Yunnan [28]. The prevalence of HAdV in Chongqing and Yunnan was at the same level.

It has been reported that HAdV infection is more common in children aged 1 to 2 years than in other age groups [23], and our study confirms this observation. Children in this age group no longer have maternal antibodies but still have an immature immune system. There was no statistical difference in the positive detection rate among different age groups in Yunnan province. The detection rate of HAdV ranged from 2.50–4.78%, which is consistent with the findings of Yang et al. [29]. HAdV was detected in all age groups. The 1- to 2-year-old age group had the highest detection rate (4.78%), followed by the 6-month- to 1-year (4.63%), and 3- to 4-year (4.35%) age groups. Overall, 84.72% of the HAdV cases were in children ≤ 2 years, and children ≤ 5 years accounted for 98.57% of all of the diarrhea cases.

The time of the HAdV infection peak can vary according to region. It was reported to be in February to March in rural Bangladesh [30]. In Konya, seasonal differences were not statistically significant for adenovirus [31], but in Thailand, HAdV infection occurred throughout the year, with a higher detection rate between May and July [23]. Our results are similar to what has been observed in Thailand. HAdV was identified throughout the year, with the highest detection rate (15.53%) in June.

In this study, the detection rate for the years 2015–2021 was 4.10%, varying from 1.03–12.26% each year. The prevalence of HAdV infection increased each year from 2015 to 2018, after which it remained at a relatively low level for two years and then increased significantly in 2021. This might be a consequence of the the prevention and control measures taken by the Chinese government against the COVID-19 epidemic.

Next-generation sequencing is a very sensitive technique that, like PCR, can be used to identify and characterize viruses that are present at a subclinical level. Thirteen complete HAdV genome sequences were determined in this study and are available in the GenBank database. The primary adenovirus antigens are the hexon, penton base, and fiber proteins [25, 32]. Analysis of the genes encoding these proteins indicated that the strains circulating in Yunnan province were closely related to those found elsewhere in China.

Sensitive surveillance systems for HAdV have not been established in the southeastern areas of China due to the slow development of technology and the economy. Continuous monitoring for HAdV is imperative for predicting the emergence of new epidemic strains and for vaccine development. To gain a better overview of the prevalence and epidemiology of HAdV, a large-scale and long-term study is needed. A nationwide epidemiological surveillance program for HAdV should be established that would allow the units responsible for disease control and prevention to conduct early detection screening and issue early warnings. In addition, efforts are needed to develop more-accurate methods to identify the major causative agents of diarrhea and to provide convenient and efficient tools for the establishment of more-effective preventive measures.
